# Assessment of Arabic Web-Based Knowledge About Root Canal Treatment: An Infodemiologic Study

**DOI:** 10.7759/cureus.59794

**Published:** 2024-05-07

**Authors:** Hatim A Qurban, Khalid N Alturki, Nouf M Alharbi, Abdullah H Alerwi, Razan J Alharbi, Muath S Alassaf

**Affiliations:** 1 Endodontics, Taibah University, Madinah, SAU; 2 Dentistry, Taibah University, Madinah, SAU; 3 Dental Education, Taibah University, Madinah, SAU; 4 Orthodontics and Dentofacial Orthopedics, Taibah University, Madinah, SAU

**Keywords:** dentistry, dental, web-based, public health, endodontics, endodontic treatment

## Abstract

Background: Root canal treatment (RCT) is a vital dental procedure aimed at preserving tooth function and minimizing infection. Access to accurate and comprehensive information about RCT is crucial for informed decision-making. With the increasing reliance on the Internet for health-related information, it is essential to evaluate the quality and readability of web-based RCT content, particularly in Arabic-speaking regions.

Methods: This study conducted an extensive web search using three major search engines (Google, Yahoo, and Bing) to identify Arabic-language websites providing information on RCT. Inclusion criteria required websites in Arabic with comprehensive RCT content. Quality assessment employed the DISCERN instrument, JAMA benchmarks, and Health on the Net (HON) assessment tools, while readability was assessed using Flesch Kincaid Grade Level (FKGL), Simple Measure of Gobbledygook (SMOG), and Flesch Reading Ease (FRE) metrics.

Results: Out of 152 websites included, the majority were affiliated with university/medical centers (56.58%) and non-profit organizations (28.29%). Quality assessment revealed that the mean DISCERN score was 2.82, indicating moderate quality. Only one website achieved a high DISCERN score. JAMA benchmarks showed limited compliance, with only two websites meeting all criteria. The HON code was found on only five websites. Readability analysis indicated that most websites were reasonably easy to read by the general population.

Conclusion: This study highlights the significant gap in the quality and reliability of web-based health information related to RCT in Arabic. While the majority of websites examined in this study did not meet established quality standards, there is a clear need for improvements in the accuracy and comprehensiveness of online resources. Patients seeking information on RCT should exercise caution and consider consulting healthcare professionals for trustworthy guidance. Further research should explore strategies to enhance the quality of web-based health information and expand the scope of evaluation to ensure that individuals have access to reliable resources for making informed decisions about their dental health.

## Introduction

Root canal treatment (RCT) is a common endodontic procedure aiming to minimize infection and preserve tooth function. It involves the removal of inflamed or necrotic pulp tissue, cleaning and shaping the root canal system, and obturation with a biocompatible material [1,2]. Procedures can range from simple RCT to endodontic surgery. Successful RCT requires proper knowledge and skills to avoid complications. However, many patients possess limited or inaccurate information about RCT, potentially affecting their decision-making and satisfaction. A previous statistic by HCUP in the United States in 2018 reported that 33.1%, 31.4%, and 32.8% of visits were due to diseases of the pulp and periapical tissues, respectively [3].

Web-based information has become a prevalent source of health-related knowledge for the public. It offers several advantages, such as convenience, accessibility, and interactivity. Such information can significantly influence patients' knowledge, attitudes, and behaviors toward their health conditions and treatments. A prior study in the US reported that 80% of the population searches the Internet for health and treatment information [4]. Meanwhile, in Saudi Arabia, a survey of 344 diabetic patients found that 39% were Internet users, and 71.6% of them used the Internet to seek health information [5].

The credibility of online information is paramount, especially considering its influence on health-related behaviors. However, the varied nature of this information, from professional guidelines to personal anecdotes, means that there is a risk of misinformation. A systematic review of the prevalence of health misinformation on social media found that some studies reported up to 87% of misinformation on certain topics, with 30% related to medical treatments [6].

Given the widespread use of the Arabic language spoken by approximately 422 million individuals and ranked as the fifth most spoken language worldwide, it is imperative to ensure that Arabic health-related information is both accurate and comprehensive. While many health domains have been thoroughly assessed for their web-based information, there remains a significant gap concerning RCT content. To date, no studies have been identified to evaluate this in either English or Arabic. Therefore, this study's primary objective is to rigorously assess the quality, accuracy, and comprehensiveness of web-based RCT content in Arabic. This research seeks to provide a benchmark for evidence-based information and guidance for Arabic-speaking individuals and professionals.

## Materials and methods

A comprehensive web search was initiated. Before delving into the search, all cookies were deleted, and incognito mode was activated to ensure unbiased search results.

Search strategy

The top three search engines, Google, Yahoo, and Bing, were employed for this study. Searches were performed using the Arabic translations for “What is root canal treatment, How RCT is performed, Causes of pulp pain” and related therapeutic interventions. The initial five result pages from each search engine were thoroughly assessed within a 24-hour timeframe to minimize the potential impact of evolving search results. Consequently, the top 50 websites from each platform for each search term were meticulously examined using the principal keyword. Throughout the search process, default settings were maintained and specialized search criteria were deliberately eschewed.

Websites faced exclusion if they were not primarily in Arabic, gave a cursory mention of RCT, were exclusively audio or visually oriented, represented in-depth scientific articles or academic textbooks, were evidently rehashed from other sources, displayed excessive ads, sponsored links, or forums, prevented access with the designated keywords, lacked substantive information on RCT, and were centered around workshops.

Inclusion criteria

Preference was given to freely accessible websites that utilized Arabic as the primary language and furnished comprehensive details about RCT.

Quality assessment

DISCERN Plus Assessment

DISCERN is a standardized tool designed to assist users in evaluating the quality of written health information on treatment choices [7]. It consists of 16 questions organized into three sections. Questions 1-8: Address the reliability of the publication, determining whether it can be depended upon as a source of information regarding a specific treatment. Questions 9-15: Concentrate on specifics of treatment options. Question 16: Gives an overall quality rating upon completion of the assessment. Each question is assessed on a five-point Likert scale where 1 suggests poor quality and 5 implies high quality. The cumulative score can offer insights into the overall trustworthiness and utility of the publication.

JAMA Benchmarks

JAMA benchmarks were introduced to help users identify the credibility of health information available online [8]. It comprises four criteria, Authorship (Information about authors, their credentials, affiliations, and potential conflicts of interest should be clear); Attribution (Clear referencing of sources or evidence on which the content is based); Currency ( Dates of content creation and updates should be available to determine the freshness of the information); Disclosure (Site ownership, sponsorship, advertising, underwriting, and commercial funding sources or potential conflicts of interest should be transparently disclosed). Although these are simple criteria, they offer a basic, yet effective, approach to assess the quality and reliability of online health information.

Health on the Net (HON) Assessment Tools

The HON Foundation promotes transparent and reliable health information online through its certification system. It contains eight primary principles, Authority (Any medical information should be provided by qualified professionals); Complementarity (Information should support, not replace, patient-physician relationships); Confidentiality (Privacy of users should be respected and maintained); Justifiability (Claims regarding benefits or performance should be backed by evidence); Attribution(Source references for health information should be clear); Financial Disclosure (Funding sources and potential conflicts of interest should be clearly stated); Transparency (Accessibility, transparency, and validity of the contact details of the health website); Advertising Policy (Clear distinctions must be made between editorial content and advertising). Both evaluators were pre-trained to ensure uniformity in the assessment process. They were both dental professionals, ensuring the evaluations were clinically relevant and accurate.

Readability assessment

We used the readability calculator website (https://readabilityformulas.com) to gauge the ease with which the website content can be read. We employed tools like Flesch Kincaid Grade Level (FKGL), Simple Measure of Gobbledygook (SMOG), and Flesch Reading Ease (FRE).

The acceptable readability level was determined as FKGL and SMOG scores less than 7, and FRE scores greater than or equal to 80.

Statistical analyses

Data were collected and recorded in Microsoft Office Excel spreadsheets (Microsoft Corporation, Redmond, USA). Descriptive and error intrareader statistical analyses were calculated using IBM SPSS Statistics for Windows, Version 26 (Released 2019; IBM Corp., Armonk, New York, United States).

Ethical considerations

In conducting this study, we have exclusively employed publicly available data that does not include personally identifiable information, confidential data, or other sensitive content related to individual patients or participants. As such, the nature of the study design doesn’t require obtaining informed consent or undergoing formal ethical review processes generally mandated for research involving human subjects.

## Results

Website availability and inclusion

**Figure 1:**
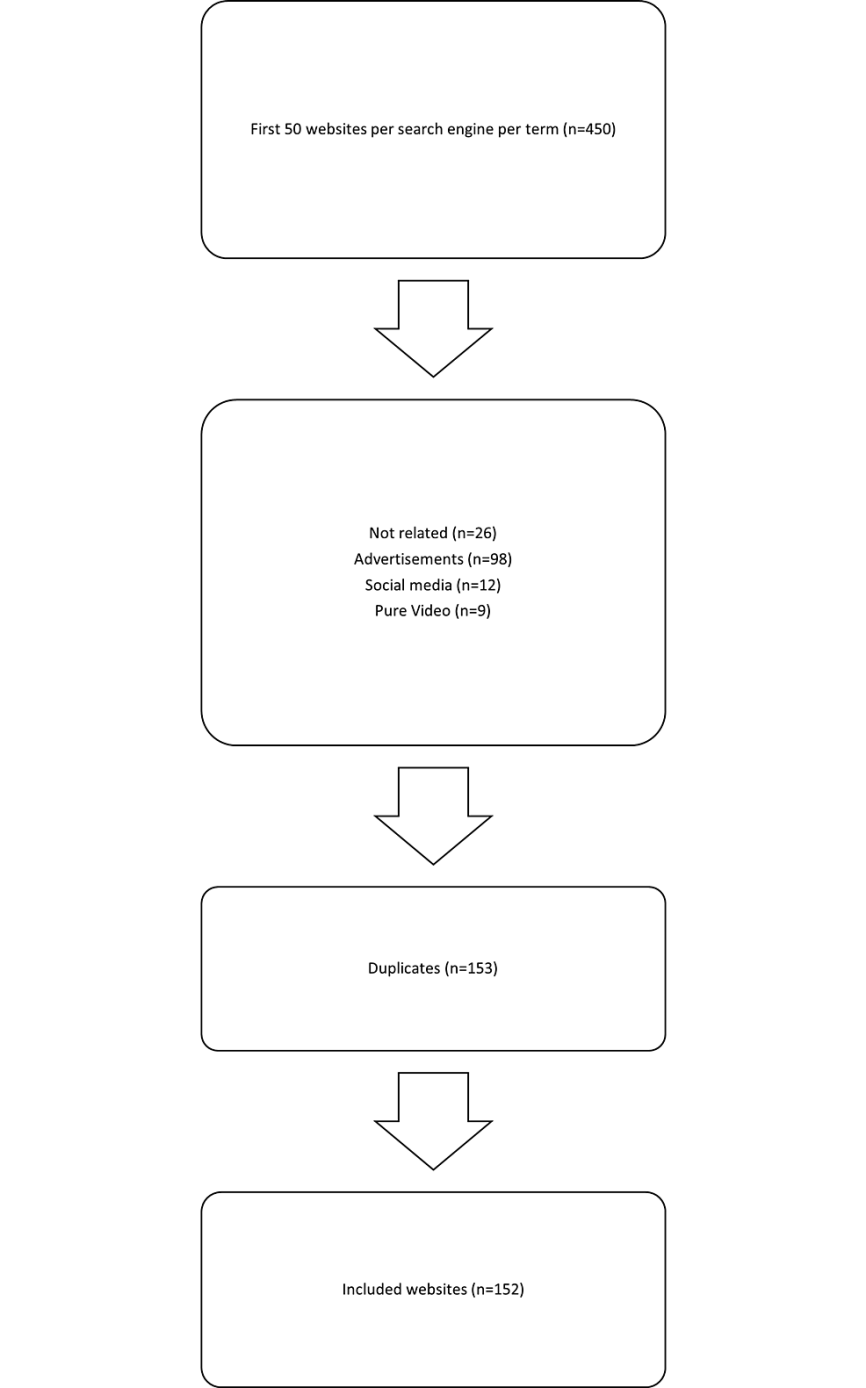
Flow chart of the search strategy for the web-based root canal treatment.

A thorough search was executed using the Arabic term for RCT, resulting in an initial dataset. After applying the inclusion criteria, a total of 152 websites were deemed eligible and were thus included in the study. This provides a comprehensive snapshot of the web-based information available on RCT treatments (Figure 1).

Website categorization

Of the 152 websites analyzed, the majority were affiliated with University/Medical Centers, accounting for 86 websites (56.58%). Non-profit organizations comprised the second-largest category, with 43 websites (28.29%). Commercial websites were less common, totaling 23 (15.13%). Governmental websites were notably absent from the sample. The dataset did not provide explicit information on the specialization of websites. This could be an area for future research (Table 1).

**Table 1:** Categorization of websites based on affiliation, specialization, content type and content presentation.

Category	Criteria	Freq.	%
Affiliation	Commercial	23	15.13
	University/medical center	86	56.58
	Non-profit organization	43	28.29
	Governmental	0	0
Specialization	Exclusively related	2	1.32
	Partly related	150	98.68
Content type	Medical facts	150	98.68
	Clinical trials	0	0
	Human interest stories	0	0
	Question and answer	88	57.52
Content presentation	Image	103	67.32
	Video	16	10.46
	Audio	0	0

Quality assessment

DISCERN Instrument

Each website was evaluated using the DISCERN instrument, which comprises 16 questions designed to assess the quality of health information. The average score across all websites was 2.82±0.69, falling under the 'Moderate' quality category. The highest score was achieved for 'Relevance' with a mean of 3.93±1.11, while 'Explicit Sources' scored the lowest with a mean of 1.75±1.30 (Table 2).

**Table 2:** Means and standard deviation scores for the quality assessment using the DISCERN instrument. Each question is assessed on a five-point Likert scale where 1 suggests poor quality and 5 implies high quality.

Domain	DISCERN question	Mean (SD)
Reliability	Q1. Explicit aims	2.59 (0.99)
	Q2. Aims achieved	2.81 (1.03)
	Q3. Relevance	3.93 (1.11)
	Q4. Explicit sources	1.75 (1.30)
	Q5. Explicit date	3.03 (1.45)
	Q6. Balanced and unbiased	3.34 (0.98)
	Q7. Additional sources	2.19 (1.12)
	Q8. Areas of uncertainty	1.95 (0.67)
Treatment options	Q9. How treatment works	3.09 (1.23)
	Q10. Benefits of treatment	2.13 (0.90)
	Q11. Risk of treatment	2.44 (1.05)
	Q12. Effects of no treatment	1.95 (1.04)
	Q13. Effects on quality of life	1.42 (0.53)
	Q14. All alternatives described	2.17 (0.95)
	Q15. Shared decision	2.14 (1.01)
Overall rating		2.82 (0.69)

JAMA Benchmarks

Regarding the JAMA Benchmarks, authorship was the most commonly met criterion, appearing on 60 websites (39.47%). Attribution was present on 37 websites (24.34%), and disclosure was the least common, found on only three websites (1.97%) (Table 3).

**Table 3:** Distribution of JAMA scores based on website affiliation as part of the quality assessment (n=152). The data in the table is presented as the number of websites (%) The "No, One, Two, Three, Four" Indicates how many criteria were achieved by each affiliation. For example, the number of non-profit organizations that achieved the three criteria of JAMA was 16 websites

Number of criteria achieved	No	One	Two	Three	Four
Commercial	4 (17.4)	6 (26.1)	8 (34.8)	5 (21.7)	0 (0.0)
Non-profit organization	1 (2.3)	3 (7.0)	21 (48.8)	16 (37.2)	2 (4.7)
University/medical Center	27 (31.4)	49 (57.0)	9 (10.5)	1 (1.2)	0 (0.0)
Total	32 (21.1)	58 (38.2)	38 (25.0)	22 (14.5)	2 (1.3)

Readability metrics

The readability of the websites was also evaluated. The average FKGL was 6.9±3.9, and the average FRES score was 94.1±10.2, indicating that most websites are reasonably easy to read (Table 4).

**Table 4:** Readability tests and their values for the web-based root canal treatment. Words and Sentences represent number of words and number of sentences in article

	FRES	FKGL	SMOG	Words	Sentences
Mean	94.1	6.9	2.2	913.1	38.7
Standard Deviation	10.2	3.9	0.7	419.2	23.9
Maximum Score	108.2	23.6	4.8	2102.5	130.0
Minimum Score	50.6	1.6	1.8	147.0	4.5

Multimedia elements

The number of multimedia elements varied across the websites. The majority of commercial websites had two to three multimedia elements, while non-profit organizations and university/medical centers mostly had one or zero.

## Discussion

The Internet has become a source of information that most people would rely on. However, questions are raised regarding the quality of this information, especially health-related ones. There is no surprise that individuals, due to the accessibility to their mobiles, would check for online information regarding their condition and symptoms. It could be argued that the availability of online information could help individuals obtain a general idea about some topics; however, for health-related information, it is always recommended to consult a doctor in that regard. 

Endodontic treatment can vary depending on the situation of the affected tooth. A study has shown that around 93% had RCT among adults [10]. Thus, it can be assumed that RCT is a common dental therapy that needs to be well demonstrated to the common individuals. On the other hand, such a high percentage would indicate that the information available to the public needs to be assessed properly to ensure that people who seek treatment can receive valid information. 

In this study, different assessment tools were used to assess the quality, the readability, and the category of the available information on the internet. The most common search engines like Google, Yahoo, and Bing were used to identify this information. It was found that the chosen questions for this study resulted in duplicate results. Thus, it can be argued that the available information is not focused on a specific topic. A recent study looked at the available information on the internet regarding post-endodontic selections, it was found that none of the websites were exclusively focused on the aimed topic [11].

The quality assessment in this study included three different tools. DISCERN, JAMA benchmark, and HONs assessment tool. The overall mean DISCERN score achieved from the assessed website was 2.82 (± 0.69). Most of the assessed websites scored low (19.74%) to moderate (79.61%) DISCERN score. However, only one website achieved a high score. Many studies reported similar results regardless of their aim of study [11-14]. This could be due to the lack of quality of information available online as they are not supported with valid evidence.

Regarding the JAMA benchmark, only two of the websites achieved the four scoring items (1.32%). On the other hand, 21.05% of the websites did not match the scoring items. 38,16% and 25% of the websites achieved one to two scoring items respectively. The HON code revealed that only five out of 152 websites had the code on their website. Those findings matched several recent studies in relation to oral health topics [13,14].

For the readability assessment, the results showed that most of the websites were reasonably easy to read as per the low score of SMOG results. Similarly, the low FKGL score indicates the understandability of the website by the common individuals. Similar results were reported by Halboub et al. in their study when they studied the health-related information available online on dental implants [15].

In terms of the categorization of the websites, the American Food and Drug Administration (FDA, 2005) stated that governmental non-profit organizations and educational institutions' websites should be considered the platforms of choice to provide reliable information. In this study, it was found that most of the websites were university/medical centers (65.58%) and non-profit organizations (28.29%). However, only two of the websites were exclusively specialized in endodontics (1.32%).

The need for the development of automated tools that regularly scan and evaluate health information on websites. These systems can utilize artificial intelligence and machine learning algorithms to assess the accuracy, reliability, and quality of content based on predetermined quality benchmarks like DISCERN and HON.

This study has several limitations as it did not evaluate the videos that appeared on the search. Also, only three search engines were implemented to achieve the results. Further research should be considered to involve more search engines and more media content.

## Conclusions

This study showed that the available data online on the topic needs to be monitored. The content available on most of the websites did not meet most of the quality assessment tools. Thus, it could be concluded that the use of the Internet to seek medical advice is not reliable and individuals need to consult a physician regarding their condition.
